# Retraction: Abscisic Acid Is Required for Root Elongation Associated With Ca^2+^ Influx in Response to Water Stress

**DOI:** 10.3389/fpls.2021.720525

**Published:** 2021-06-23

**Authors:** Frontiers Editorial Office

The Journal and Authors retract the 22 April 2020 article cited above for the following reasons provided by the Authors:

In April 2020, the article “Abscisic Acid Is Required for Root Elongation Associated With Ca^2+^ Influx in Response to Water Stress” was published in *Frontiers in Plant Science*. Concerns were subsequently raised regarding the provenance of some materials used in the study. Specifically, during the investigation, the authors failed to provide appropriate authorization for use of the seeds of the UBQ10-GcAMP6S transgenic line.

This retraction was approved by the Chief Editors of *Frontiers in Plant Science* and the Editor-in-Chief of Frontiers. The authors accept their responsibility in this case.

